# Membranous CD24 drives the epithelial phenotype of pancreatic cancer

**DOI:** 10.18632/oncotarget.9402

**Published:** 2016-05-17

**Authors:** Clara Lubeseder-Martellato, Ana Hidalgo-Sastre, Carolin Hartmann, Katharina Alexandrow, Zahra Kamyabi-Moghaddam, Bence Sipos, Matthias Wirth, Florian Neff, Maximilian Reichert, Irina Heid, Günter Schneider, Rickmer Braren, Roland M. Schmid, Jens T. Siveke

**Affiliations:** ^1^ II. Medizinische Klinik und Poliklinik, Klinikum Rechts der Isar, Technical University Munich, Munich, Germany; ^2^ Institute of Radiology, Klinikum Rechts der Isar, Technical University Munich, Munich, Germany; ^3^ Institute of Pathology, University Tübingen, Tübingen, Germany; ^4^ Current address: Klinik für Anaesthesiologie, Klinikum Rechts der Isar, Technical University Munich, Munich, Germany; ^5^ German Cancer Consortium (DKTK) and German Cancer Research Center (DKFZ), Heidelberg, Germany; ^6^ Division of Gastroenterology, Perelman School of Medicine, University of Pennsylvania, Philadelphia, PA, USA; ^7^ Division of Solid Tumor Translational Oncology, West German Cancer Center, University Hospital Essen, Essen, Germany

**Keywords:** CD24, pancreatic ductal adenocarcinoma, β-catenin, MET, EMT

## Abstract

Surface CD24 has previously been described, together with CD44 and ESA, for the characterization of putative cancer stem cells in pancreatic ductal adenocarcinoma (PDAC), the most fatal of all solid tumors. CD24 has a variety of biological functions including the regulation of invasiveness and cell proliferation, depending on the tumor entity and subcellular localization. Genetically engineered mouse models (GEMM) expressing oncogenic *Kras^G12D^* recapitulate the human disease and develop PDAC. In this study we investigate the function of CD24 using GEMM of endogenous PDAC and a model of cerulein-induced acute pancreatitis. We found that (i) CD24 expression was upregulated in murine and human PDAC and during acute pancreatitis (ii) CD24 was expressed exclusively in differentiated PDAC, whereas CD24 absence was associated with undifferentiated tumors and (iii) membranous CD24 expression determines tumor subpopulations with an epithelial phenotype in grafted models. In addition, we show that CD24 protein is stabilized in response to WNT activation and that overexpression of CD24 in pancreatic cancer cells upregulated *β-catenin* expression augmenting an epithelial, non-metastatic signature. Our results support a positive feedback model according to which (i) WNT activation and subsequent β-catenin dephosphorylation stabilize CD24 protein expression, and (ii) sustained CD24 expression upregulates β-catenin expression. Eventually, membranous CD24 augments the epithelial phenotype of pancreatic tumors. Thus we link the WNT/β-catenin pathway with the regulation of CD24 in the context of PDAC differentiation.

## INTRODUCTION

CD24 is a mucin-like glycosylphosphatidylinositol (GPI)-anchored protein, which was originally discovered as a leucocyte expressed ligand for P-selectin [1-3]. Several studies show enhanced CD24 expression in various cancers including PDAC, where CD24 expression correlates with advanced disease stages [1-4]. In PDAC, cells expressing surface CD24, CD44 and ESA were identified as putative cancer stem cells [5, 6]. CD24 expression also regulates cell motility and invasion [7-10]. In particular, it was reported that CD24 expression in intracellular vesicles inhibits cell invasion in pancreatic cancer cells [11]. Overall, there is strong evidence for a pivotal role of CD24 in tumor cell migration and cell proliferation. However, CD24 function depends on the tumor entity and subcellular localization. The role of plasma membrane-bound CD24 in PDAC development remains unclear, yet is of particular interest, since surface CD24 expression serves as a cancer stem cell marker [6-13].

Epithelial-mesenchymal transition (EMT) plays a key role in the regulation of cell motility and invasion, during this process the disassembly of adherens junctions is required during EMT [14]. Thus, one hallmark of EMT is the dissociation of the E-cadherin/β-catenin complex from adherens junctions. β-catenin is not only present at the plasma membrane, a second cytoplasmic pool of β-catenin is phosphorylated by a destruction complex containing GSK3 and constitutively degraded *via* ubiquitination in the proteasome [15]. Upon activation of the WNT pathway the destruction complex dissociates from β-catenin and allows the accumulation of a hypophosphorylated form of β-catenin in the cytosol [16], which eventually enters the nucleus and activates transcription [15-18].

In this study, we focus on the function of CD24 in genetically engineered mouse models (GEMM)-based endogenous PDAC and in cerulein-induced experimental acute pancreatitis. We observe that increased intracellular CD24 expression correlates with cytoplasmic β-catenin expression *in vivo*. Using xenograft models, we demonstrate that surface CD24 is required for the development of differentiated tumors. We show that activation of the WNT pathway followed by β-catenin dephosphorylation stabilizes intracellular CD24 protein and that CD24 overexpression regulates both mesenchymal and epithelial markers. Thus we propose, a novel crosstalk between the WNT/β-catenin pathway and the regulation of CD24 in the context of PDAC differentiation.

## RESULTS

### CD24 expression in human and mouse PDAC

**Figure 1:**
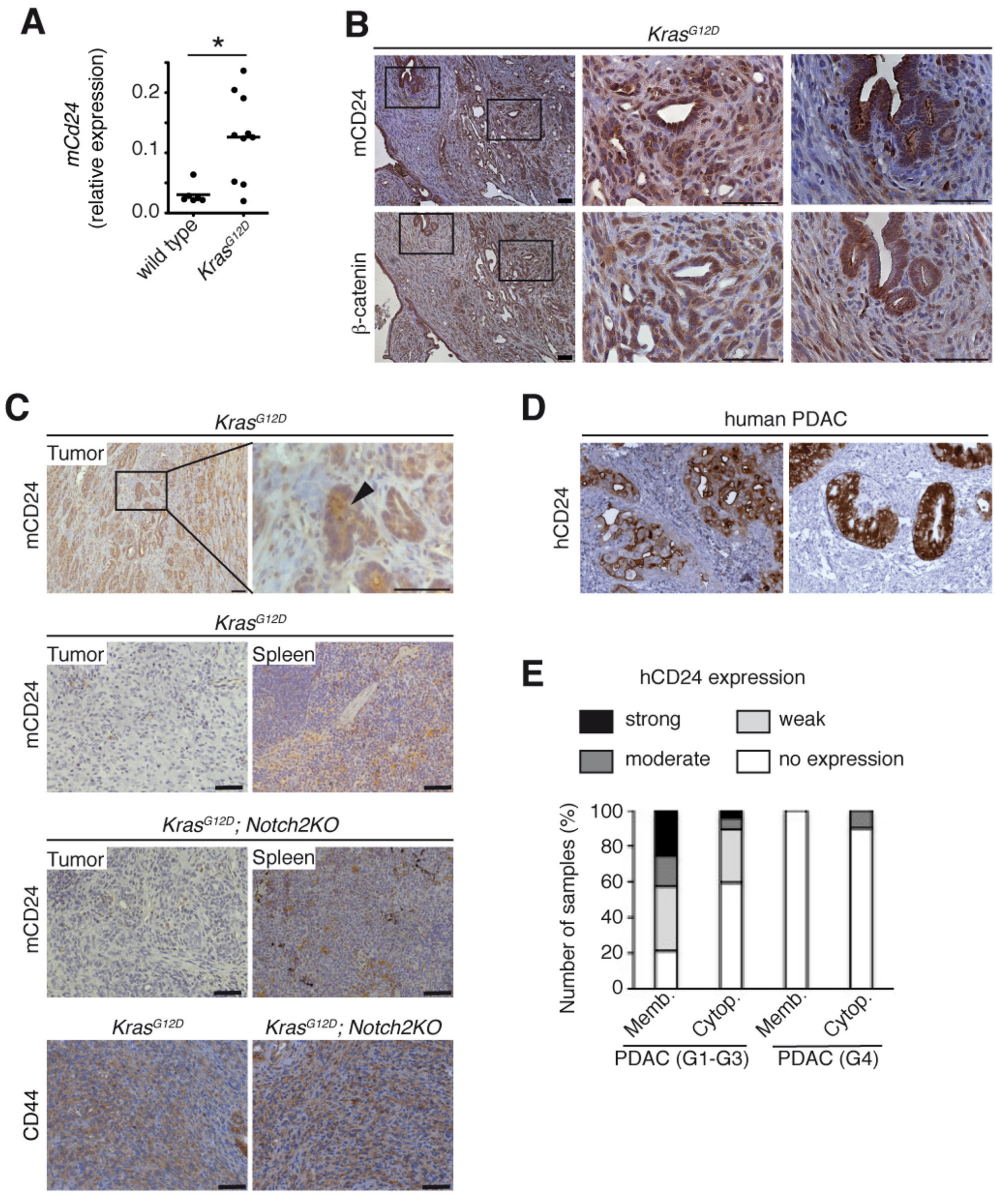
h/mCD24 is expressed in differentiated PDAC **A.** *Cd24* mRNA expression increased in *Kras^G12D^* mice compared to wild type at the age of 6 months. **B.** Immunohistological staining for mCD24 and β-catenin in murine differentiated PDAC. **C.** Immunohistological staining of murine differentiated and undifferentiated PDAC of indicated genotypes. Spleens from the same mice were used as a positive control for staining. **D.** Immunohistological staining for hCD24 in human PDAC. Examples showing membranous, cytoplasmic or both staining patterns. **E.** A series of human ductal (G1-G3) and undifferentiated (G4) pancreatic cancers was stained for hCD24. The membrane-bound and cytoplasmic stainings were scored according to a three-tiered system (1 - <10%, 2 - 11-50%, 3 - >50% of the cells are positive). Scale bars = 50 μM.

*Ptf1a^+/Cre(ex1)^*;Kras*^+/LSL-G12D^* mice (referred to as *Kras^G12D^*), develop preneoplastic lesions that progress to invasive tumors recapitulating human PDAC [19]. Expression of *mCd24* was significantly increased in pancreata of *Kras^G12D^* mice at the age of 6 months (Figure 1A). CD24 expression was both intracellular and membranous in pancreatic acini and PanIN lesions of *Kras^G12D^* mice (Supplementary Figure S1). In tumor cells, CD24 was expressed in the cytoplasm of integrin-β3-negative cells (Supplementary Figure S2A, S2B). Remarkably, in *Kras^G12D^*-driven tumors CD24 was strongly expressed in ductal lesions in concomitance with cytoplasmic β-catenin expression, while it was absent in undifferentiated tumors (Figures 1B, 1C and Supplementary Figure S1A). Previous studies have shown that *Kras^G12D^* activation with concomitant pancreas-specific deletion of *Notch2* (referred to as *Kras^G12D^*;*Notch2KO* hereafter) leads to increased epithelial-mesenchymal transition (EMT) [20, 21]. While we observed strong CD24 expression in well-differentiated tumors, CD24 expression was absent in undifferentiated tumors from both *Kras^G12D^* and *Kras^G12D^*;*Notch2KO* mice, which all expressed CD44 (Figure 1C). Expression of further cancer stem cell markers like CD133 and Nestin was unaffected (Supplementary Figure S1B). Of note, metastatic lesions of *Kras^G12D^*;*Notch2KO* were more differentiated compared to the primary tumors and re-expressed CD24 (Supplementary Figure S1C). Confocal analysis of *Kras^G12D^* pancreata revealed a vesicular staining pattern of CD24 in pancreatic acinar cells and PanIN lesions in agreement with published data (Supplementary Figure S1D) [11]. Notably the CD24-positive vesicles partially co-localized with β-catenin and E-cadherin at the plasma membrane (Supplementary Figure S1D, arrows). These results correlate CD24 expression with the epithelial phenotype of differentiated tumors.

In order to correlate hCD24 expression to clinicopathological data, we next evaluated hCD24 protein expression in human PDAC samples (N=57) (Figure 1D, 1E). Membranous hCD24 expression was observed in 37 of 47 PDAC (78%; 17 weak, 8 moderate, 12 strong), while cytoplasmic hCD24 staining was more infrequent (40%; 14 weak, 3 moderate, 2 strong). When the tumors were dichotomized for no/weak vs. moderate/strong hCD24 expression and analyzed for survival, there was no difference in survival of patients (membranous hCD24, log rank test, p=0.714; cytoplasmic hCD24 expression, log rank test, p=0.252). Undifferentiated PDAC (G4) are considered to involve EMT of tumor cells. In agreement with the expression pattern observed in the mouse model, only one of ten G4 PDACs expressed membranous hCD24 (Figure 1E); intracellular staining was not observed in these tumors.

### Membranous mCD24 leads to differentiated tumors in xenografts

Next, we screened murine cell lines derived from *Kras^G12D^* (N= 7) and *Kras^G12D^*;*Notch2KO* (N=7) PDAC by FACS analysis and 85.7% of the cell lines expressed mCD24 (Supplementary Figure S2C). Although undifferentiated tumors derived from *Kras^G12D^*;*Notch2KO* mice did not express mCD24 as described above, 6 out of 7 cell lines from *Kras^G12D^*;*Notch2KO* mice re-expressed mCD24. This observation suggests that there is a survival advantage for cells expressing mCD24 in culture. To further address this issue, we investigated hCD24 expression in a 3D organoid model from human PDAC as recently described [22]. As shown in Supplementary Figure S2D, organoids derived from human PDAC did not express hCD24 in 3D culture, while they express E-cadherin as expected [22]. When organoids were seeded on plastic dishes using 2D culture conditions, all independent organoids (N = 3) readily re-expressed hCD24 (Supplementary Figure S2D, lower panel).

**Figure 2:**
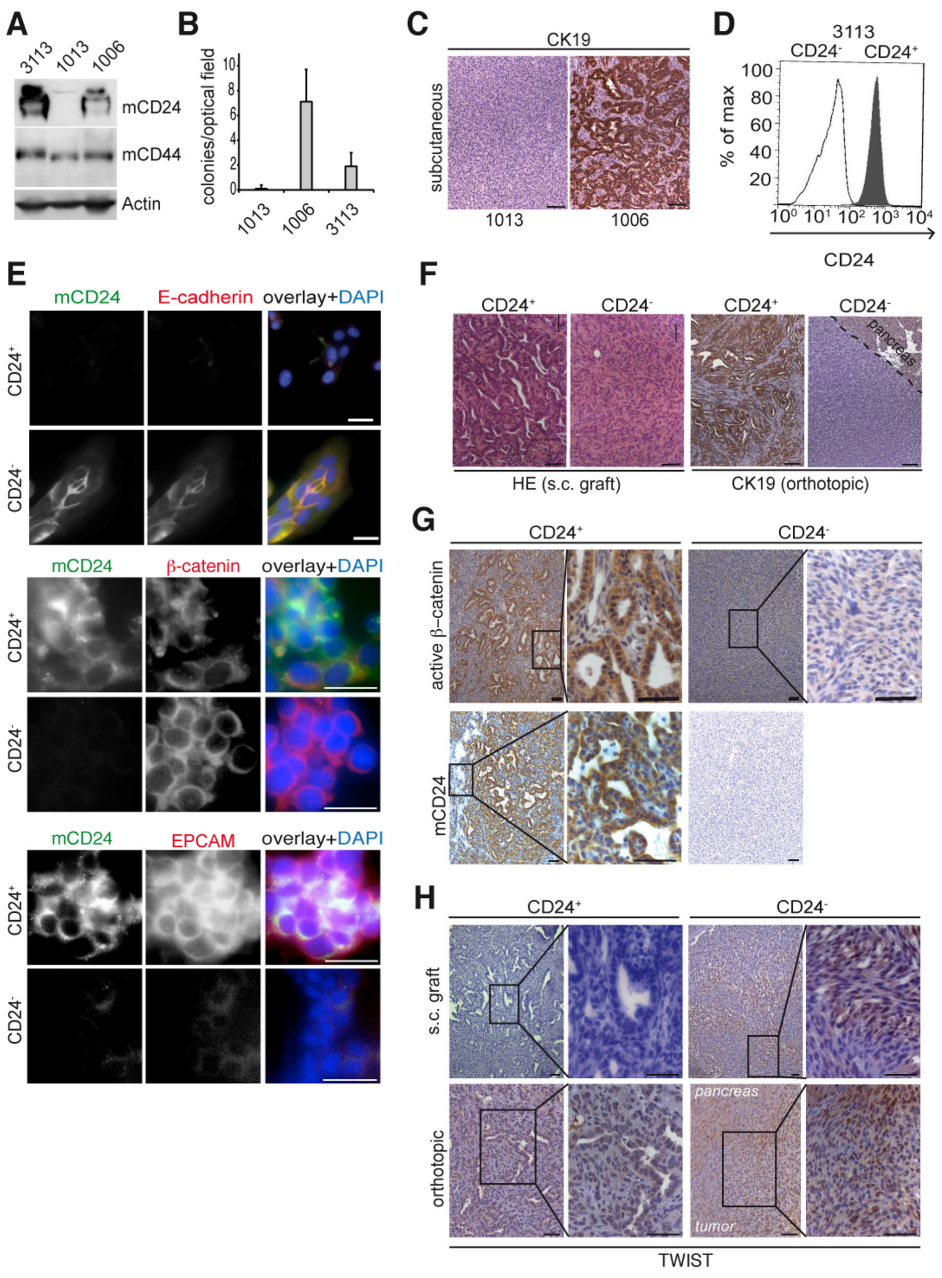
mCD24 expressing tumor cells lead to differentiated tumors Pancreatic cells were either mCD24 negative (#1006) or mCD24 positive (#1006 and #3113). **A.** Western blot analysis of primary mouse cell lines. **B.** Soft agar assay. Mean ± SD. **C.** immunohistological analysis of s.c. tumors generated from CD24^−^ or CD24^+^ pancreatic cells. **D, E.** Pancreatic cells (#3113) were sorted into CD24^−^ and CD24^+^ cell populations. D, purity of sorted populations was confirmed by FACS analysis using a mCD24-FITC antibody. E, immunofluorescence staining of sorted cells. **F.** Left panels: histology of tumors (N=13) generated by s.c. injections of CD24^−^ and CD24^+^ pancreatic cells. Right panels: immunohistological analysis for CK19 expression of tumors generated by orthotopic transplantation of CD24^−^ and CD24^+^ pancreatic cells. **G, H.** Immunohistological analysis of s.c. and orthotopic tumors, as described in F, showing strong cytoplasmic expression of active β-catenin in CD24+ tumors and nuclear TWIST expression in CD24- tumors. Scale bars = 50 μm.

Next, we selected three *Kras^G12D^*;*Notch2KO* derived cell lines with different mCD24 expression levels: CD24 positive (#1006), CD24 negative (#1013) or mixed CD24 expression (#3113) (Figure 2A and Supplementary Figure S2C). Expression of the surface marker mCD44 was equal in the three cell lines (Figure 2A and Supplementary Figure S2C). mCD24 positive cells showed reduced migratory ability (Supplementary Figure S2E), which is in agreement with previous studies [11] and were characterized by β-catenin localization at cell-cell contacts (Supplementary Figure S2F) in concomitance to β-catenin phosphorylation and GSK3 de-phosphorylation at S9 (Supplementary Figure S2G). Only the cell lines expressing mCD24 were able to form colonies in a soft agar assay (Figure 2B), suggesting that mCD24 expression is required for anchorage-independent growth. Because anchorage-independent growth is a hallmark of tumorigenicity, we next investigated the ability of mCD24 positive and negative tumor cells to form tumors in syngenic C57BL/6 mice. Both CD24 positive and CD24 negative cells generated tumors after approximately 3 weeks (Figure 2C). Tumors generated by CD24 positive cells were characterized by the presence of many well-differentiated duct-like lesions expressing CK19. In contrast, tumors generated by CD24 negative cells were undifferentiated (Figure 2C). These results suggested that surface mCD24 may be a regulator of the epithelial phenotype.

To further confirm our results we sorted cells by FACS from the cell line described above (#3113) to get isogenic CD24^+^ and CD24^−^ cell populations (Figure 2D). Both cell populations expressed β-catenin, while the CD24^+^ population only expressed E-cadherin and EPCAM (Figure 2E). Both CD24^+^ and CD24^−^ cells led to tumor formation after subcutaneous injection (Figure 2F). However, differentiated tumors with ductal lesions arose only from CD24^+^ tumor cells and these tumors only expressed active β-catenin (Figures 2F, 2G). By contrast, undifferentiated tumors arose only from the CD24^−^ cell population and were characterized by expression of the mesenchymal transcription factor TWIST (Figure 2H). Similar results were obtained when CD24^+^ and CD24^−^ sorted cells where orthotopically transplanted in the pancreas of syngenic C57BL/6 mice (Figure 2H, lower panels). Accordingly, tumors that originated from the CD24^+^ cells were characterized by strong E-cadherin expression and absence of TWIST (Figure 2H and Supplementary Figure S2H) thus supporting their epithelial phenotype. Taken together these experiments demonstrate that surface mCD24 expression of pancreatic tumor cells is a key feature for the development of an epithelial phenotype.

### mCD24 expression in cerulein-induced acute pancreatitis

**Figure 3:**
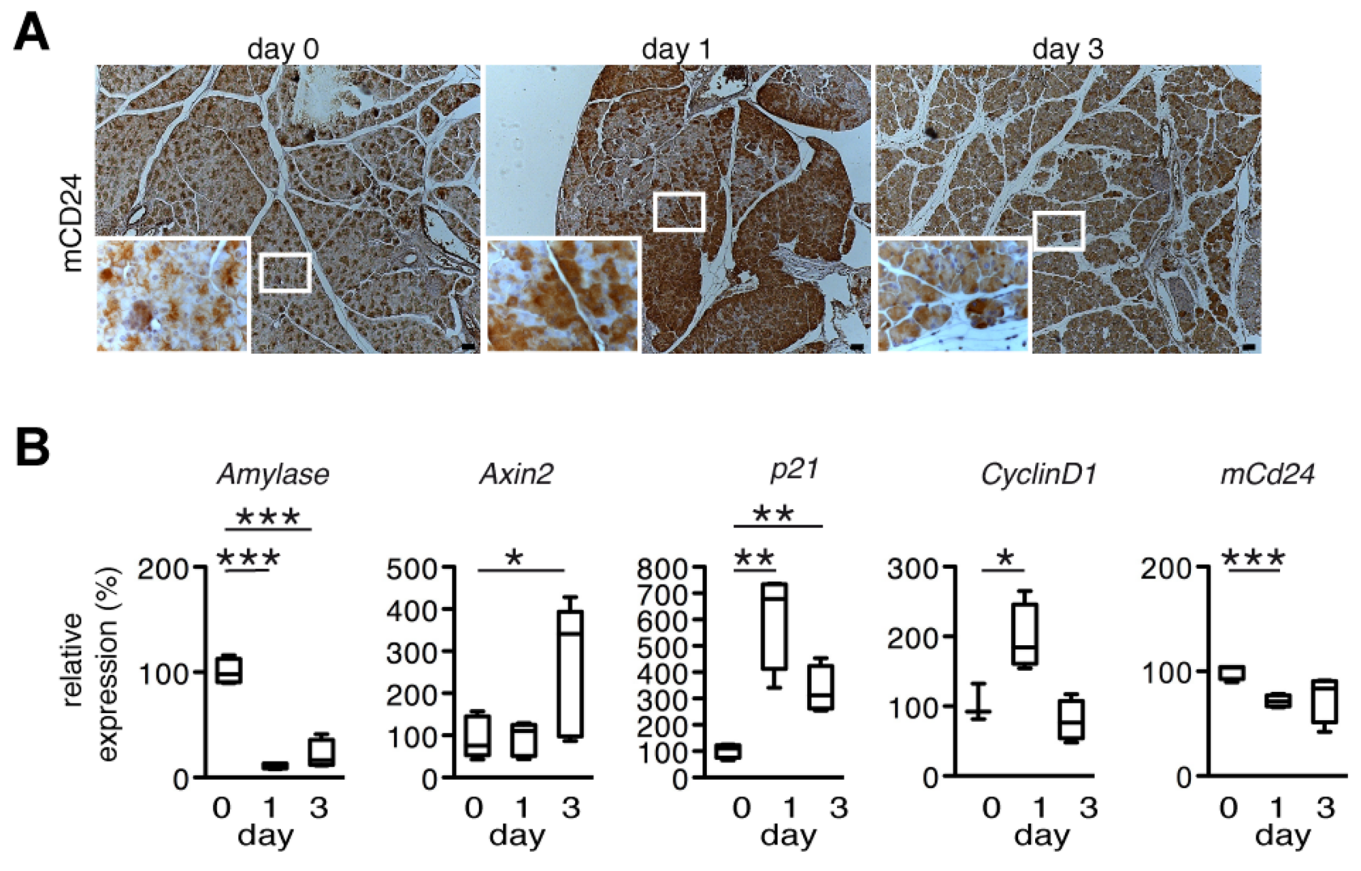
mCD24 expression in a mouse model for acute pancreatitis **A.** Murine pancreatic tissue of an experimental model for cerulein induced acute pancreatitis was analyzed by immunohistochemistry at the indicated time points. mCD24 expression was strongly upregulated at day 1. Scale bars = 50 μm. **B.** Pancreatic tissue from the same mice as in A was lysed and relative expression of the indicated genes was analyzed by real-time qRT-PCR.

Having analyzed mCD24 expression in PDAC models, we wondered if a correlation exists between mCD24 expression and β-catenin during acute pancreatitis as the WNT/β-catenin pathway is activated during this process [23]. Acute pancreatitis was induced in mice by hourly intraperitoneal cerulein injections (Supplementary Figure S3A). Acinar genes, such as *Amylase* were downregulated, indicating successful induction of pancreatitis (Figure 3B). The β-catenin target genes *Axin2*, *p21* and *CyclinD1* were upregulated, indicating activation of the WNT/β-catenin signaling pathway. However, *mCd24* expression was downregulated at day 1 and did not change at day 3 (Figure 3B). By contrast, mCD24 protein expression was strongly increased in pancreatic acini in concomitance with sustained β-catenin cytoplasmic expression (Figure 3A and Supplementary Figure S3B). These results suggest that accumulation of intracellular β-catenin stabilizes mCD24 protein expression.

Notch1-dependent β-catenin transcriptional activity is inhibited by treatment with the γ-secretase inhibitor dibenzazepine (DBZ) during acute pancreatitis [24]. β-catenin was expressed in the cytoplasm of pancreatic acini of mice treated with DBZ at day 3 of experimental acute pancreatitis in concomitance with high intracellular mCD24 expression (Supplementary Figure S4B). Thus, not the transcriptional activity of β-catenin, but rather its cytoplasmic localization seems to be the key property for sustained intracellular mCD24 expression.

### WNT activation and CD24 protein expression

**Figure 4:**
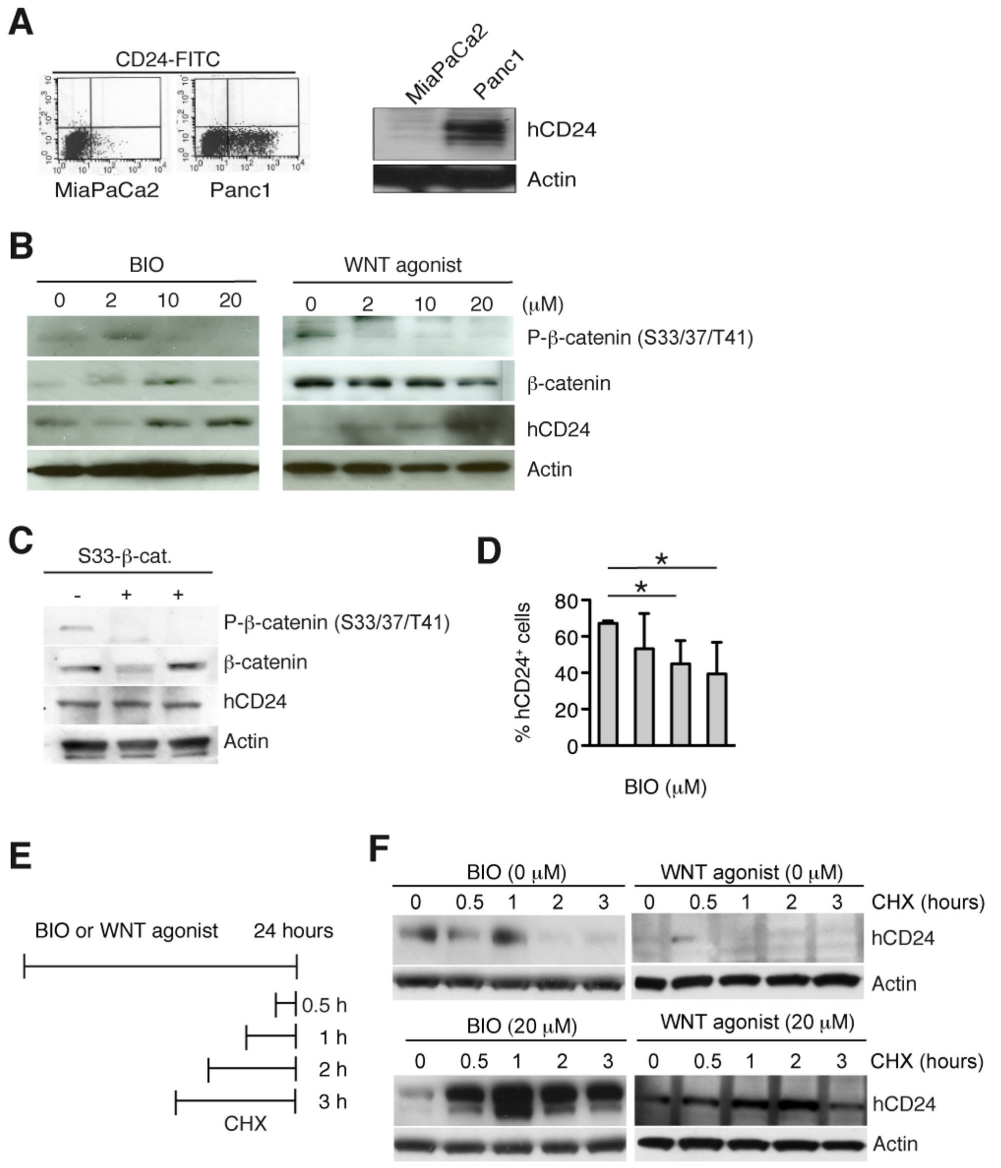
Activation of canonical WNT pathway increases hCD24 expression by stabilization of CD24 protein **A.** FACS analysis of surface (left panel) and total (right panel) hCD24 expression in human PDAC cell lines. **B.** Panc1 cells were stimulated with BIO or WNT agonist for 24 hours. Western blot analysis shows a decrease in β-catenin phosphorylation indicating successful activation of the WNT pathway in concomitance with an increase in hCD24 expression. **C.** Panc1 cells were transiently transfected with an expression vector containing active β-catenin (S33-β-catenin). hCD24 expression was not affected. **D.** FACS analysis for hCD24 surface expression in Panc1 cells after stimulation with BIO. Mean ± SD. **E.** Panc1 cells were treated with BIO or WNT agonist for 24 hours and cycloheximide (CHX) was added before harvesting the cells at the indicated time points. **F.** Panc1 cells were treated as described in D: Western blot analysis shows increased hCD24 expression.

Next we used two different cell permeable activators of the WNT pathway. Both WNT agonist [25] and the GSK3β–inhibitor BIO [26] induce dissociation of β-catenin from the destruction complex and its dephosphorylation at serine 33, 37 and threonine 41 (S33/37/T41). We first tested hCD24 expression in two human cell lines as shown in Figure 4A and used Panc1 cells that express about 50% hCD24 for further experiments. Treatment of human and murine PDAC tumor cells with WNT agonist and BIO led to dephosphorylation of β-catenin and increased m/hCD24 protein expression in a dose-dependent manner (Figure 4B and Supplementary Figure S5A). Expression of *mCd24* RNA was unaffected while *Cdh1* was downregulated (Supplementary Figure S5B) under these various conditions. To test whether transcriptionally active β-catenin is required for hCD24 upregulation, a constitutively active form of β-catenin (S33-β-catenin) was transfected in Panc1 cells. While S33-β-catenin did not affect hCD24 protein expression (Figure 4C), activation of WNT signaling by pharmacological inhibition of GSK3β decreased surface hCD24 localization (Figure 4D) and induced intracellular hCD24 accumulation (Supplementary Figure S5C). Next, we inhibited *de novo* protein synthesis by treatment of pancreatic cells with cycloheximide (CHX) at different time points in presence or absence of the WNT activators (Figure 4E). CHX treatment increased hCD24 expression at all time points (Figure 4F). Taken together, these results show that WNT activation stabilizes intracellular m/hCD24 protein expression independently of β-catenin transcriptional activity.

The WNT/β-catenin signaling pathway is involved in epithelial plasticity, including both EMT and MET. TGFβ-induced EMT is controlled by IKKα in Panc1 cells [27]. TGFβ treatment of epithelial PDAC tumor cells reduced membranous hCD24 expression in concomitance to loss of E-cadherin expression (Supplementary Figure S6A). Conversely, stable transfection of mesenchymal tumor cells with an IKKα-specific shRNA [27] led to an epithelial phenotype and sustained CD24 expression (Supplementary Figure S6B, S6C). IKKα-transfected cells upregulated the hypophosphorylated form of β-catenin, this is consistent with studies showing a role of IKKα in regulating β-catenin phosphorylation [28]. These results not only suggest that induction of EMT downregulates mCD24 expression, but are also in agreement with the restriction of m/hCD24 expression to differentiated tumors.

### hCD24 regulates MET markers

**Figure 5:**
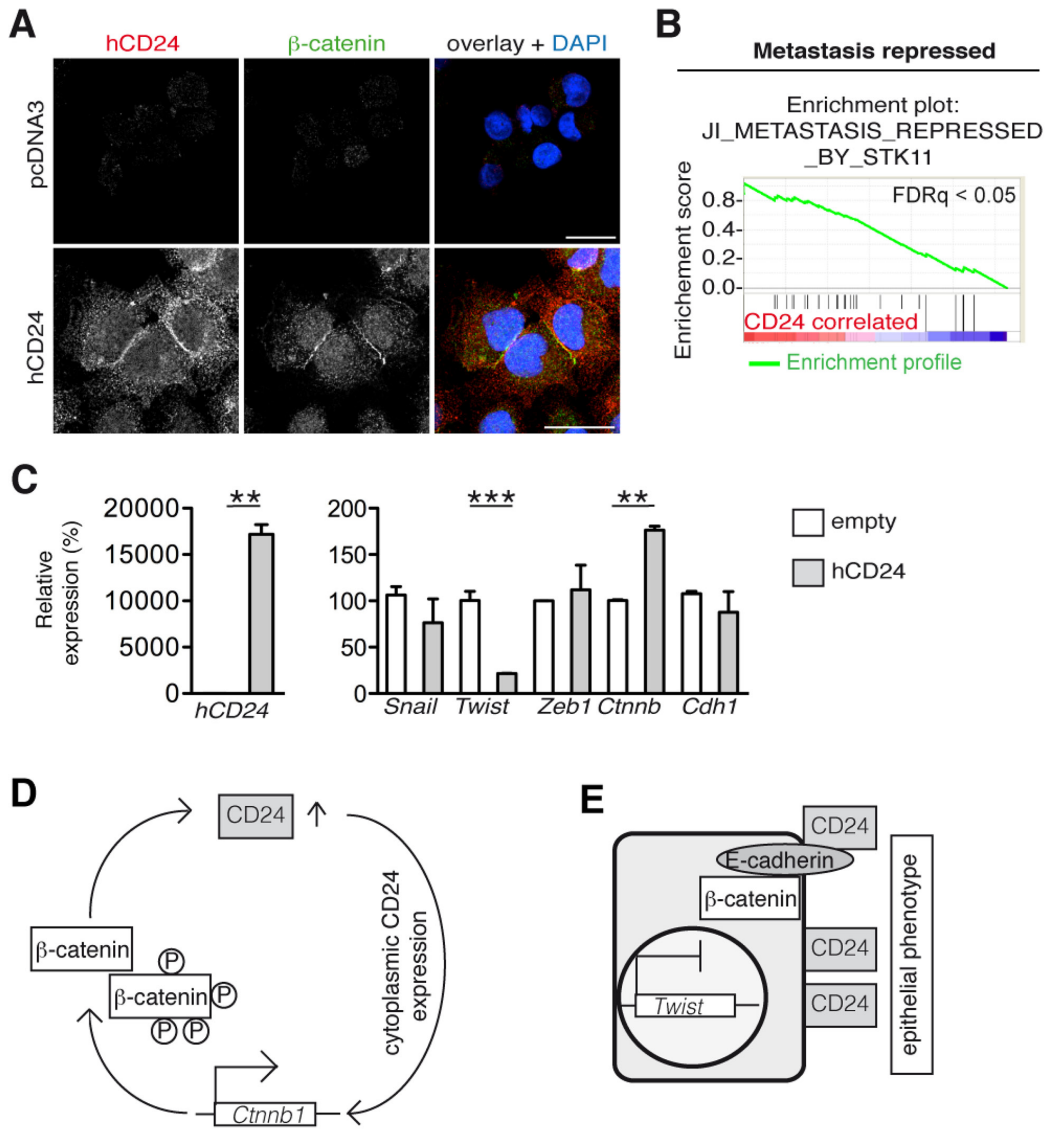
hCD24 regulates MET **A.** The mesenchymal cell line MiaPaCa2 was transiently transfected with an hCD24 expression vector. Cells were fixed after 48 hours and analyzed by immunofluorescence. Cells expressing hCD24 had an epithelial phenotype characterized by membranous β-catenin expression. **B.** Gene set enrichment analysis of pcDNA3-CD24 transfected MiaPaCa2 cells. 24 hours after transfection mRNA was isolated and analyzed using an Ilumina beadchip. Statistical analyses: FDRq < 0.05, nominal p-value < 0.05. **C.** MiaPaCa2 cells were transiently transfected with hCD24 for 24 hours. Left panel shows successful transfection. Right panel: analysis of mesenchymal transcription factors by real-time qRT-PCR. Mean ± SD. **D.** Working model: after an initial increase of CD24 expression in the pancreas, WNT signaling leads to β-catenin dephosphorylation and to a further increase of CD24 protein. Sustained CD24 expression upregulates β-catenin expression. **E.** Eventually CD24 accumulation at the cell surface stabilizes an epithelial phenotype.

We further investigated the mechanism involved in hCD24 expression and regulation of the epithelial phenotype. The PDAC cancer cell line MiaPaCa2 harbors mesenchymal features and hardly expressed hCD24 compared to Panc1 cells, which have a more ductal phenotype (Figure 4A and Supplementary Figure S6D). We transiently transfected MiaPaCa2 cells with a hCD24 expression plasmid (Figure 5A). hCD24 expressing cells acquired expressed membranous β-catenin (Figure 5A). Next, we performed microarray-based transcriptional profiling of transfected MiaPaCa2 cells using gene set enrichment analysis (GSEA). CD24 overexpression correlated with repression of genes involved in metastasis (Figure 5B) that is linked to EMT. When we analyzed single genes involved in MET regulation by real time qRT-PCR, we found that the mesenchymal transcription factor *Twist* was downregulated while the epithelial marker *Ctnnb* was upregulated in concomitance with *hCd24* overexpression (Figure 5C). Thus hCD24 is a regulator of epithelial-mesenchymal transition phenotypes.

## DISCUSSION

In this study we show that CD24 expression in the genetic *Kras^G12D^*-based murine PDAC model is comparable to the human disease. CD24 has been shown to be a key player in several types of tumors, sometimes with conflicting results depending on CD24 subcellular localization [8, 11]. Intriguingly, we observe strong intracellular CD24 expression in concomitance with cytoplasmic β-catenin both in PDAC and acute pancreatitis mouse models. Cytoplasmic, dephosphorylated β-catenin is indicative of active WNT signaling. Upon binding of WNT to its ligands, GSK3β and the destruction complex are recruited to the membrane, thus allowing accumulation of cytoplasmic dephosphorylated β-catenin that eventually translocates to the nucleus for transcriptional activation of target genes [18, 29-31]. Further, we show that activation of the WNT pathway stabilizes intracellular CD24 protein in pancreatic tumor cells, and that inhibition of GSK3β has the same effect, while transcriptional activation of β-catenin is not required in this process. Thus, it is likely that enrichment of the β-catenin cytoplasmic pool is required for increased CD24 stability. Such hypothesis is further supported by the upregulation of CD24 during acute pancreatitis in concomitance to inhibition of Notch1-dependent β-catenin transcriptional activity by DBZ treatment. Although further cell biological studies will be required to elucidate the mechanism of the WNT activation on CD24 stability, this result is of interest because the β-catenin pathway is a key regulator of acinar cell plasticity and plays a role both in oncogenic *Kras*-driven PDAC and in acute pancreatitis [23, 32].

Expression of CD24 in tumor cells correlates with inhibition of metastatic gene signatures, downregulates the mesenchymal transcription factor *Twist* and upregulates β-catenin expression. Intriguingly, cells expressing membranous CD24 display β-catenin localization at the plasma membrane as part of adherens junctions, thus linking membranous CD24 expression with an epithelial phenotype. The involvement of CD24 in the stabilization of an epithelial phenotype during pancreatic tumor development is supported by our *in vivo* experiments demonstrating that cells expressing surface CD24 generate exclusively differentiated tumors expressing β-catenin and lacking TWIST expression. In addition, the observation that CD24 is mainly expressed in the cytoplasm of tumor cells in endogenous GEMMs suggests that surface CD24 expression is transient during PDAC development. This transient surface localization could also explain the small number of tumor cells expressing surface CD24 that can be sorted from human tumors [6]. Our observation that undifferentiated tumors do not express CD24, while differentiated metastasis of *Kras^G12D^*;*Notch2KO* mice re-express CD24 supports the hypothesis that MET is required for metastatic colonization [35]. Thus, the regulation of CD24 expression during metastatic seeding and growth underlies a dynamic and multilayered regulation. Several studies attribute different functions to CD24 depending on its subcellular localization and it has previously been proposed that intracellular CD24 inhibits tumor cell migration by regulation of BART [11]. It is intriguing to imagine that surface CD24 “traps” tumor cells in an epithelial phenotype thus inhibiting development to a mesenchymal phenotype, a prerequisite for tumor cell migration [33].

Several studies propose that both EMT and MET are relevant biological processes during development of metastasis and they have been linked to cancer stem cell (CSC) properties [34, 35-37]. CD24 and CD44 are reported CSC marker for PDAC [6]. In this context, CD24 was absent in undifferentiated PDAC of both *Kras^G12D^* and *Kras^G12D^*;*Notch2KO* mice, while the expression of CD44 and other CSC markers showed no differences. Thus, the studied CSC markers were independent of the different genetic background of mice. Based on the observation that tumor cells in hepatic metastasis expressed CD24 despite absent expression in the primary tumor, we speculate that while tumor cells undergo EMT or develop mesenchymal features, CD24 expression is downregulated. This hypothesis is supported by our observation that TGFβ-dependent EMT reduces CD24 expression.

Altogether, these observations highlight that CD24 subcellular localization is a key feature for eliciting different effects depending on the context, although the posttranslational modifications and other molecular differences, such as the sequence between intracellular CD24 and cell surface CD24 are still unknown. In summary, our results suggest that CD24 acts as an effector of the WNT/β-catenin pathway required for tumor differentiation, and we propose a positive feedback model summarized in Figure 5D-5E. First, oncogenic *Kras^G12D^* induces *Cd24* mRNA expression, which is in agreement with studies showing CD24 as a target of KRAS [38]. Second, activation of the WNT pathway and cytoplasmic accumulation of β-catenin stabilize CD24 at the protein level. Sustained CD24 expression leads to further upregulation of β-catenin and downregulation of the mesenchymal transcription factor *Twist*. Eventually CD24 accumulation at the cell surface stabilizes an epithelial phenotype. Alterations in β-catenin expression are common in pancreatic cancer and correlate with tumor differentiation, highlighting the importance of our findings [39-41]. A future challenge will be to understand the mechanistic underpinnings and factors that regulate the localization and translocation of CD24 within the cellular compartments, which may lead to novel therapeutic strategies targeting tumor differentiation.

## MATERIALS AND METHODS

### Mouse strains and experimental pancreatitis

*Ptf1a^+/Cre(ex1)^*; Kras*^+/LSL-G12D^*; Notch2*^flox/flox^* mouse strains have been described before [19-21, 23, 40]. Mice were of mixed 129SV/C57BL/6 background. Animal experiments were conducted in accordance with the German Federal Animal Protection Laws and were approved by the Institutional Animal Care and Use Committees of the Technical University Munich.

### Subcutaneous transplantation of sorted cells into SCID mice

C.B-17/Icr (SCID) mice from Charles River were bred and maintained under specific pathogen-free conditions. Sorted cells were suspended in 10 μl of DMEM. For xenografts 10^3^ or 10^4^ cells were injected subcutaneously in each flank of mice of 8 weeks of age (N=13). After 29 to 69 days of tumor growth, animals were sacrificed, and tumors were removed, paraffin-embedded and subjected to immunohistochemical analysis. For orthotopic transplantation, 10^4^ cells were injected directly into the pancreas of adult wild type animals (N=2).

### Induction of acute pancreatitis

Experimental induction of acute pancreatitis was performed by cerulein treatment essentially as described [24].

### Cell culture and biological reagents

Primary mouse pancreatic cancer cells, Panc1 and MiaPaCa2 cells were cultured in DMEM or RPMI media. The IKKα overexpressing stable MiaPaCa2 clone was produced by Günter Schneider [27]. All media were supplemented with 10% fetal calf serum. GSK inhibitor, BIO (6-bromoindirubin-3′-oxime, stock solution 10 mM in DMSO) and WNT agonist (2-Amino-4-(3,4-(methylenedioxy)benzylamino)-6-(3-methoxyphenyl)pyrimidine, stock solution 10 mM or 20 mM in DMSO) were purchased from Calbiochem.

### Cell biological assays

For soft agar assay, 6-well tissue culture plates were coated with 0.5% agar (Biozym) in DMEM. Cells to be assayed were resuspended at 37°C in 0.35% agar in DMEM. After solidification of agar, full medium was added on the top. 5000 cells were seeded per well in triplicates. After two weeks plates were stained with crystal violet and colonies were counted in 10 optical fields/well. For the invasion assay, growth factor reduced matrigel invasion chambers with a pore diameter of 8 μM (BD Biosciences) were used.

### Immunohistochemistry and immunofluorescence

Immunoperoxidase staining was performed on 4% PFA/PBS fixed paraffin-embedded tissue slides using the Vectastain ABC Elite kit (Vector Labs) following manufacturer's instructions.

For immunofluorescence staining, cryosections (6 μM) or cells grown on glass chamber slides were fixed with PFA 2% for 20 min and permeabilized with Triton X-100 0.1 % for 5 minutes. Primary antibodies were incubated overnight at 4°C and secondary antibodies (Alexa dyes, Invitrogen) for 1 h at room temperature. Sections were mounted with Vectashield hard mounting medium containing DAPI (Vector Laboratories) and examined using a Zeiss inverted fluorescent microscope or with a Leica TCS SP5 confocal microscope and the Leica Application Suite (LAS) software for acquisition. Images were processed using ImageJ software [42].

### Immunohistochemistry of human samples

Paraffin embedded tissue blocks of ductal and undifferentiated pancreatic carcinomas were routinely processed for immunohistochemistry. PDAC cases have been selected to provide a maximal statistic power regarding survival. From a large cohort of patients, short (<12 month) and long (> 36 month) survivors were chosen. In order to minimize the effect of confounders, stage T3, M0, and R0 cases were included in this analysis. The use of human tissues for research purposes was approved by the local ethics committee at the University Hospital Tübingen (number: 470/201BO1).

Following heat mediated antigen retrieval in a pressure cooker, immunohistochemical staining was performed with a mouse monoclonal anti-CD24 antibody (10 μg/ml, Clone SN3b, Thermo Scientific, Waltham. MA, USA) on a Tecan immunostainer. Detection was performed by polyvalent peroxidase–based polymer system (Zymed, Berlin, Germany). One pathologist (B.S.) evaluated the membrane-bound and cytoplasmic staining by using a three-tiered scoring system (1 - <10%, 2 - 11-50%, 3 - >50% of the cells are positive).

### Western blotting

For Western blot analysis of CD24 expression, cells were lysed in a membrane protein buffer containing Tris-Hcl (pH8) 50 mM, NonitedP40 0.5 %, SDS 0.1%, NaCl 150 mM, EDTA 1 mM and supplemented with complete protease and phosphatase inhibitors (Roche). Protein lysates were separated on a 7.5-15% polyacrylamide-SDS gel, transferred to PVDF membranes and blocked in skimmed milk followed by antibody incubation overnight at 4°C. Antibody binding was visualized using horseradish peroxidase-labeled secondary antibodies and ECL reagent (Amersham).

### Transfections and qRT-PCR

Transfections and qRT-PCR were performed using standard procedures; for detailed description and primers see the Supplementary Methods.

### GSEA

Microarraybased transcriptional profiling and analysis using RNA from three independent transfection experiments was performed using the DKFZ Genomics and Proteomics Core Facility. The HumanHT-12 v4 Expression BeadChip Array (Illumina) was used according to the manufacturer's protocols. GSEA software was provided by the Broad Institute of the Massachusetts Institute of Technology and Harvard University, Cambridge, MA (http://www.broad.mit.edu/gsea/) [43, 44].

### Flow cytometry and sorting

Cells were detached from culture plates by incubation with Trypsin-EDTA. After washing, cells were stained with the antibody of interest or matched isotype controls for 15 min at 4°C. After two washes cells were analyzed using FACS Calibur Flow Cytometer (BD). Cell sorting was performed on a FACSAdria cell sorter from BD and analyzed with the software FACS Diva 6.1.1. Experiments were repeated 3 times and the purity of sorted populations varied between (95-98%).

### Antibodies

The hCD24 (FL4/80), hCD24 (for ICH), CD24 (3H1143), mCD24 (M 1/69), CD44 (#550538), Nestin (#556309) mCD24-FITC, β-catenin, P-β-catenin (S33/37/T41) (#9561), active b-catenin, E-cadherin, actin, cytokeratin 19 (CK19), HSP90 antibodies were all purchased from BD Biosciences. The CD133 (NB120-16518) antibody was purchased from Novus Biological.

### Statistical analyses

For all analyses, the unpaired two-tailed *Mann-Whitney* test was performed with *P* < 0.05 considered significant.

### Human organoids

The use of human PDAC tissue for research purposes was approved by the local ethics committee at the Klinikum rechts der Isar (Project-Number 207/15). Generation and culturing of organoids was performed as recently described [22]. Organoids were stained as previously described in Nature protocols [45].

### Abbreviations

ESA, epithelial specific antigen; PDAC, pancreatic ductal adenocarcinoma; GEMM, genetically engineered mouse models; TGFβ, tumor growth factor beta; ADM, acinar-ductal metaplasia; EMT, epithelial-mesenchymal transition; MET, mesenchymal-epithelial transition; CSC, cancer stem cells.

## SUPPLEMENTARY MATERIALS FIGURES


