# Supplementary material for: Membranous CD24 drives the epithelial phenotype of pancreatic cancer
Source: Oncotarget. 2016 May 17;7(31):49156–68. doi: 10.18632/oncotarget.9402 (PMC5226498; doi:10.18632/oncotarget.9402)
Supplement: Supplementary file 1 [file oncotarget-07-49156-s001.pdf]

SUPPLEMENTARY METHODS

Transfections

Cells were seeded on a 6 well plate. The next day, cells were transfected using 2 µg of hCD24 and 4 µl of lipofectamine 2000 (Invitrogen) with Opti-MEM medium (Gibco), according to manufacturer instructions. The cell culture medium was changed 4 hr after transfection and cells were lysed 24 hours post-transfection, using 100 µl of RA lysis buffer (Macherey-Nagel).

RNA extraction and qRT-PCR

Total RNA was extracted from cells using the Maxwell 16 LEV simply RNA kit (Promega), according to manufacturer's instructions. A total of 2 µg of RNA was reverse transcribed to cDNA, in 20 µl reactions, using Super Script II RT (Applied Biosystems) and following manufacturer's instructions. The resultant cDNA was diluted 10-fold and 5 µl was used as template for quantitative real time PCR (qRT-PCR). qRT-PCR was performed in triplicate, in a total volume of 20 µl, using the primers listed below, and 480 SYBR Green I Master (Roche) according to manufacturer's instructions. The relative amount of each gene of interest was normalized to the reference gene GAPDH using the ΔΔCT method (Livak, K. J. & Schmittgen, T. D. Analysis of relative gene expression data using real-time quantitative PCR and the 2(-Delta Delta C(T)) Method. (*Methods* 25, 402-408, 2001)).

Primer list for qRT-PCR experiments

Primer name	Sequence
RT-hCtnnB_F	AGCTGACCAGCTCTCTCTTCA
RT-hCtnnB_R	CCAATATCAAGTCCAAGATCAGC
RT-hSnail1_F	GCACATCCGAAGCCACAC
RT-hSnail1_R	GGAGAAGGTCCGAGCACA
RT-hCdh1_F	CCCGGGACAACGTTTATTAC
RT-hCdh1_R	GCTGGCTCAAGTCAAAGTCC
RT-hCd24_F	GACATGGGCAGAGCAATGGTGGC
RT-hCd24_R	GAGTGAGACCACGAAGAGACTGGC
RT-hTwist1_F	TCGGACAAGCTGAGCAAGAT
RT-hTwist1_R	CTCCATCCTCCAGACCGAG
RT-hZeb1_F	TCAAAAGGAAGTCAATGGACAA
RT-hZeb1_R	GTGCAGGAGGGACCTCTTTA
RT-hGapdh_F	GCTCTCTGCTCCTCCTGTTC
RT-hGapdh_R	ACGACCAAATCCGTTGACTC
RT-CyclophilinD1 F	ATGGTCAACCCCACCGTG
RT-CyclophilinD1 R	TTCTGCTGTCTTTGGAACTTTGTC
RT-Amylase F	TGGTCAATGGTCAGCCTTTTTC
RT-Amylase R	CACAGTATGTGCCAGCAGGAAG
RT-Axin2 F	GCCAATGGCCAAGTGTCTCT
RT-Axin2 R	GCGTCATCTCCTTGGGCA
RT-p21 F	CACAGCGATATCCAGACATTCAG
RT-p21 R	CGGAACAGGTCGGACATCA
RT-CyclinD1 F	CTGTGCCACAGATGTGAAGTTC
RT-CyclinD1 R	AGTCCGGGTCACACTTGATGA
RT-mCd24 F	GCTCCTACCCACGCAGATTTA
RT-mCd24 R	CCCTCTGGTGGTAGCGTTACTT

SUPPLEMENTARY FIGURES

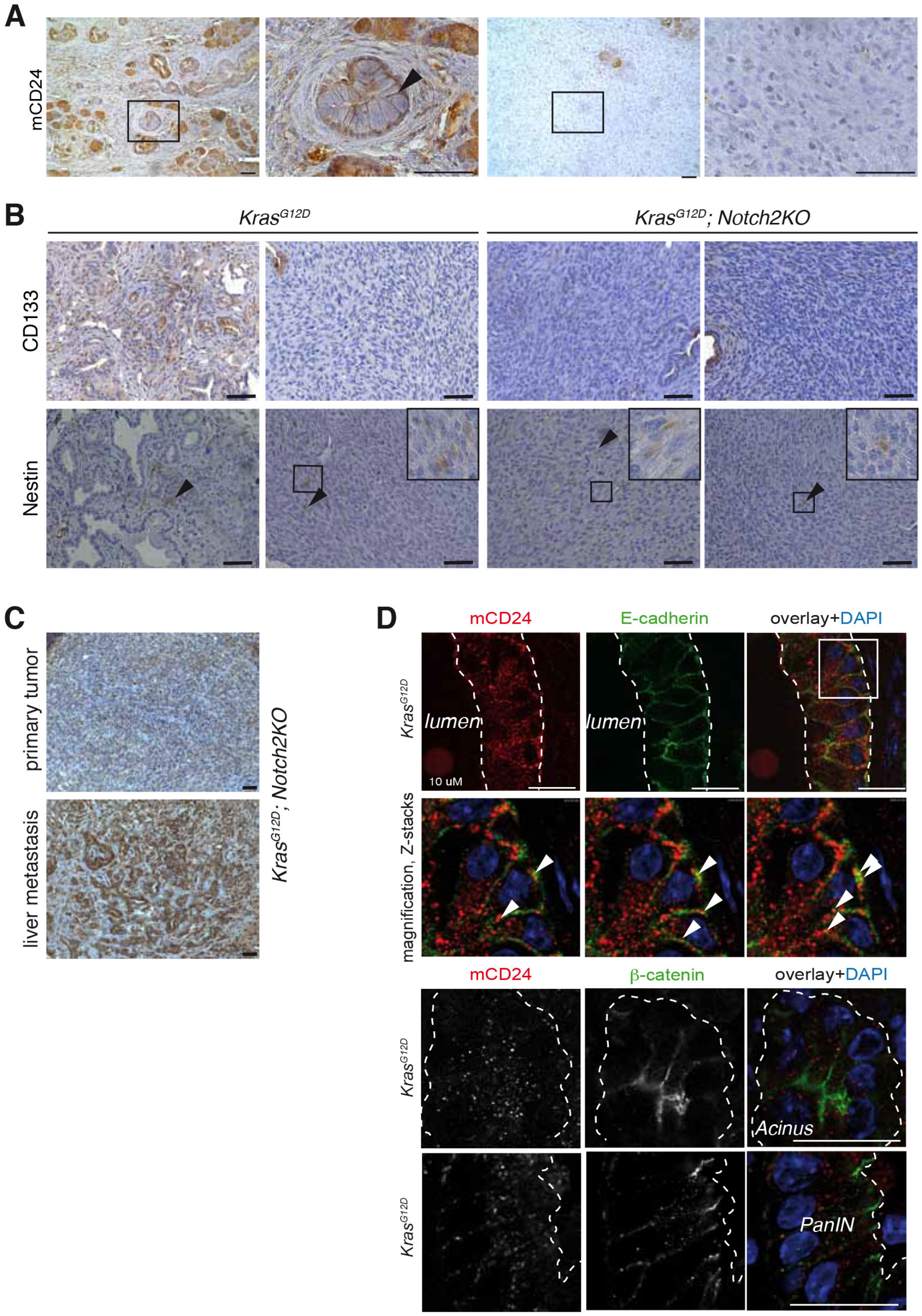

Supplementary Figure S1: CD24 localization. A. Immunohistological staining for mCD24 in pancreatic sections of *Kras*^{G12D} mice. Scale bars = 50 µM. **B.** Immunohistological staining for cancer stem cell markers in pancreatic sections of mice of the indicated genotypes. Scale bars = 50 µM. **C.** Immunohistological staining for mCD24 of a primary tumor and matched liver metastasis. **D.** Confocal analysis shows vesicular localization of CD24 in the pancreas of 3-6 months old *Kras*^{G12D} mice. Part of CD24+ vesicles colocalize with E-cadherin and β-catenin at adherent junctions (arrows). Images of the single channels were taken sequentially. Scale bars = 10 µM.

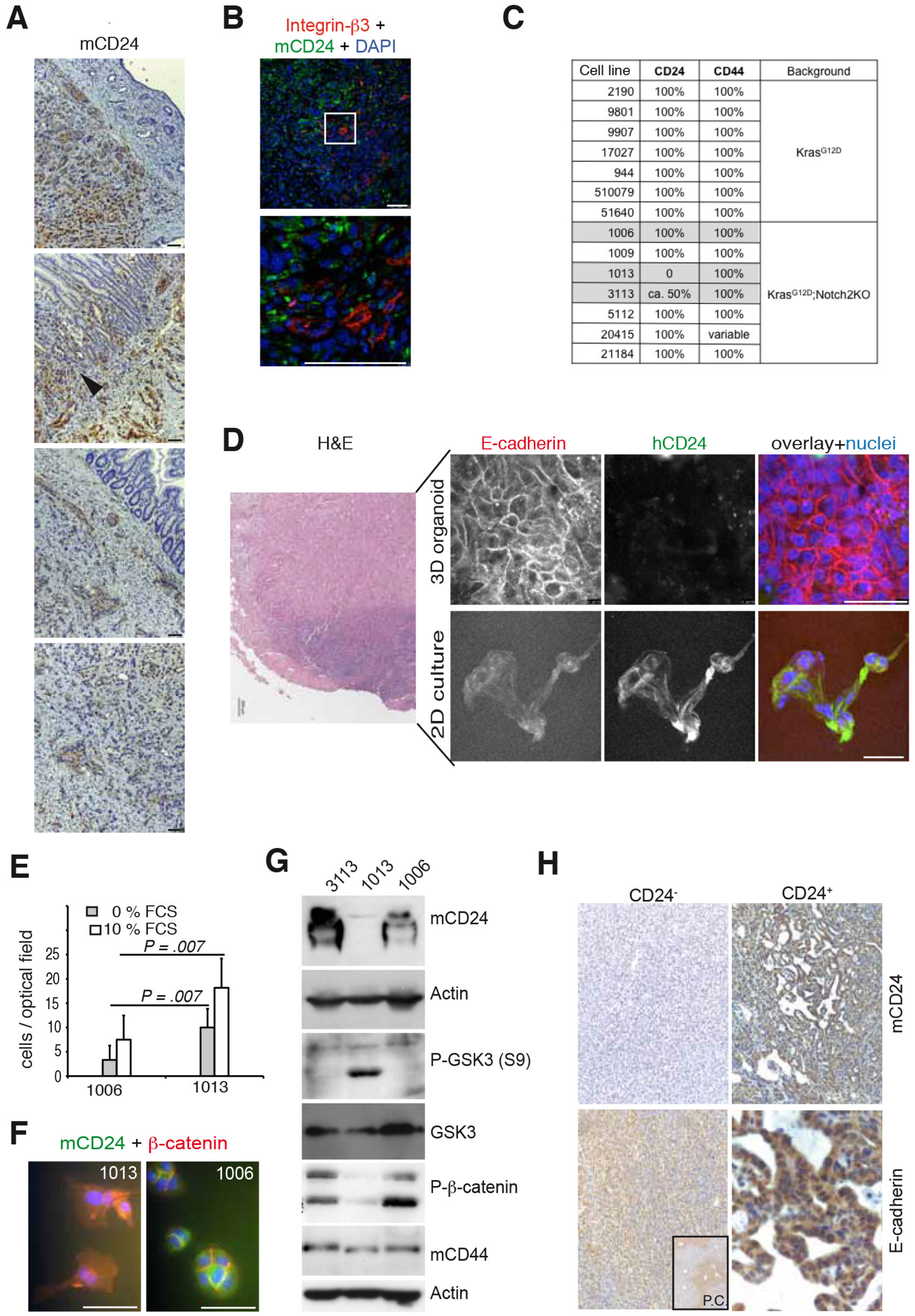

Cell line	CD24	CD44	Background
2190	100%	100%	$Kras^{G12D}$
9801	100%	100%	
9907	100%	100%	
17027	100%	100%	
944	100%	100%	
510079	100%	100%	
51640	100%	100%	
1006	100%	100%	$Kras^{G12D}$;Notch2KO
1009	100%	100%	
1013	0	100%	
3113	ca. 50%	100%	
5112	100%	100%	
20415	100%	variable	
21184	100%	100%	

Supplementary Figure S2: CD24 expression. A. Immunohistological staining in tissue sections from murine invasive PDAC. **B.** Integrin-β3 and mCD24 immunofluorescence staining of murine invasive PDAC. **C.** Tumor cells derived from murine PDACs were analyzed for CD24 and CD44 expression by FACS analysis. **D.** Organoids were generated from human tissue and cultivated in a 3-dimensional matrigel matrix (3D) or on conventional 2 dimensional palstic dish (2D). H&E staining of the original tissue. 3D and 2D cultures were analyzed by immnofluorescence staining. Scale bars = 50 µM. **E.** migration assay. Cells that were either CD24 positive (#1006) or CD24 negative (#1013). Mean ± SD. Quantification of migrated cells was performed 48 hours later. **F.** Immunofluorescence staining of murine pancreatic tumor cell lines. **G.** Western blot analysis of murine pancreatic tumor cell lines. **H.** Immunohisochemistry of orthotopic tumors. Scale bars = 50 µM.

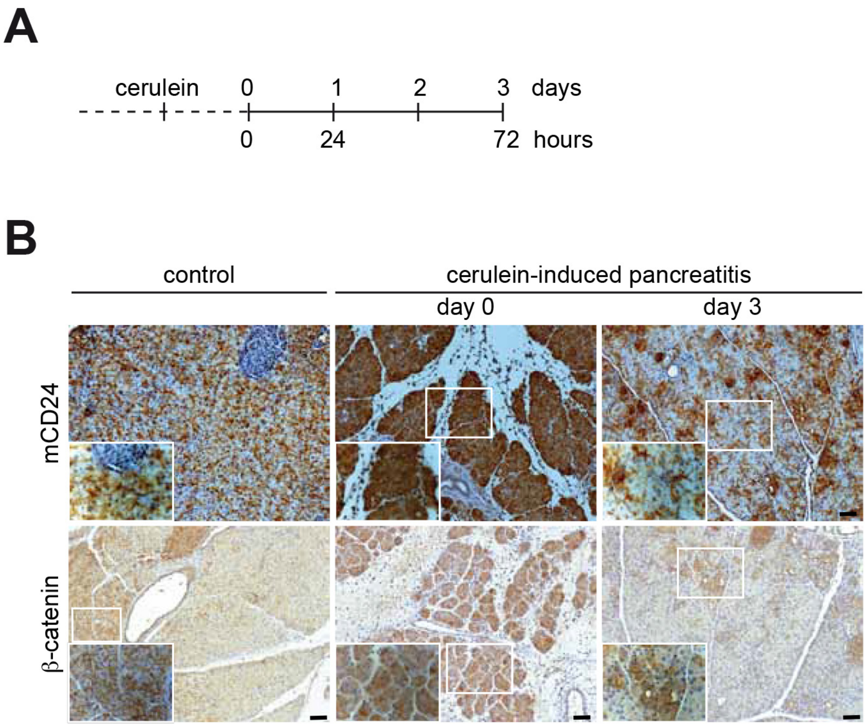

Supplementary Figure S3: CD24 expression in a mouse model for acute pancreatitis. A. Experimental design used for the induction of acute pancreatitis. **B.** Pancreatic tissue of mice treated as described in A was stained for mCD24 and β-catenin. CD24 expression was strongly upregulated after induction of acute pancreatitis in concomitance with increased cytoplasmic β-catenin expression. Scale bars = 50 μM.

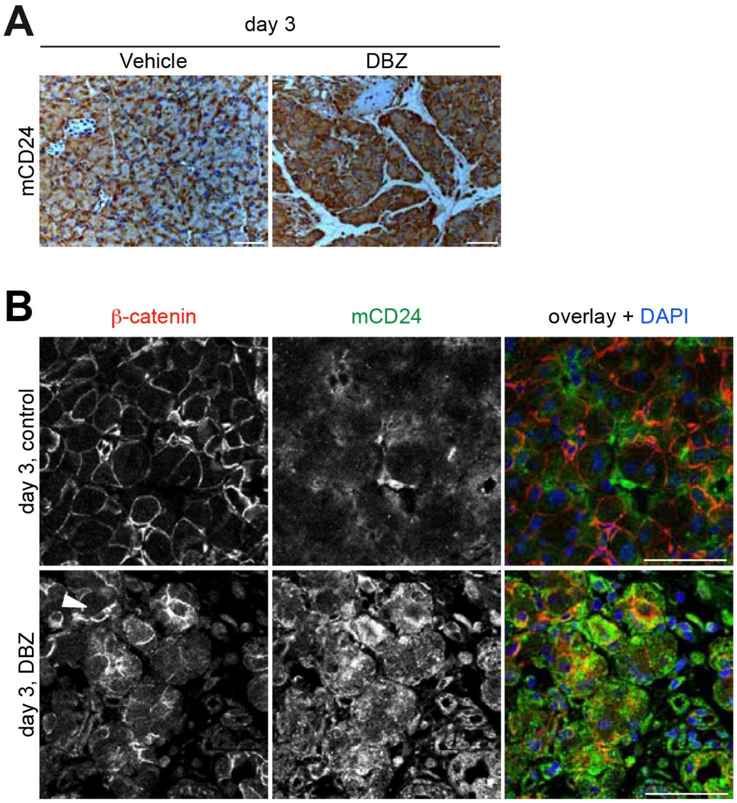

Supplementary Figure S4: CD24 expression in a mouse model for acute pancreatitis treated with DBZ. A, B. Acute pancreatitis was induced in wild type mice. Inhibition of γ-secretase with DBZ strongly enhanced CD24 expression. B, confocal pictures of immunofluorescence stainings. Mice were treated as described in A. Images of the single channels were taken sequentially. Scale bars = 50 μM.

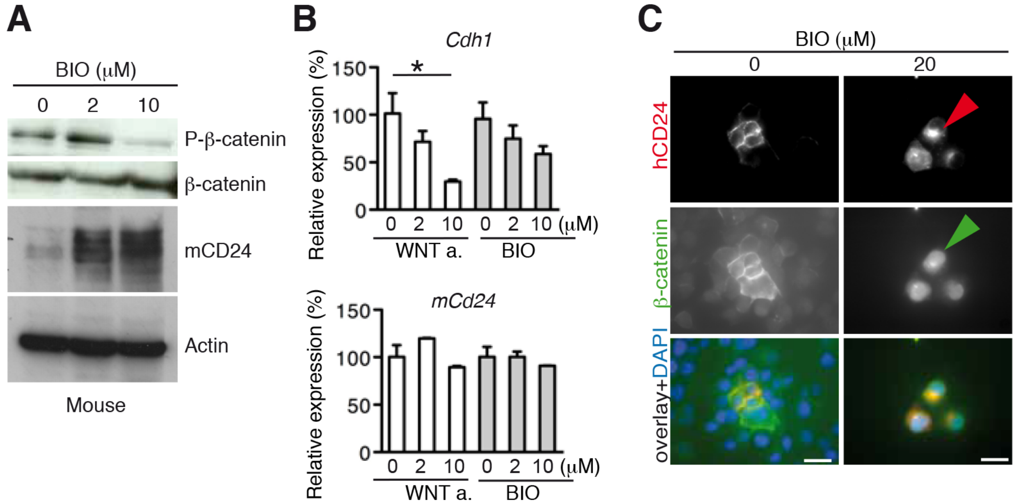

Supplementary Figure S5: Activation of the WNT pathway. A. Murine pancreatic cells (#1006) were stimulated with BIO for 24 hours. Western blot analysis shows a decrease in β-catenin phosphorylation (S33/37/T41) indicating successful activation of the Wnt pathway in concomitance with increase of CD24 expression. **B.** Pancreatic cells were treated as described in A and real-time qRT-PCR analysis for the indicated genes was performed. **C.** pancreatic cells were treated with BIO for 24 hours as described above, immunofluorecence staining shows intracellular accumulation of CD24 (red arrow) after β-catenin activation (green arrow). Scale bars = 50 μM.

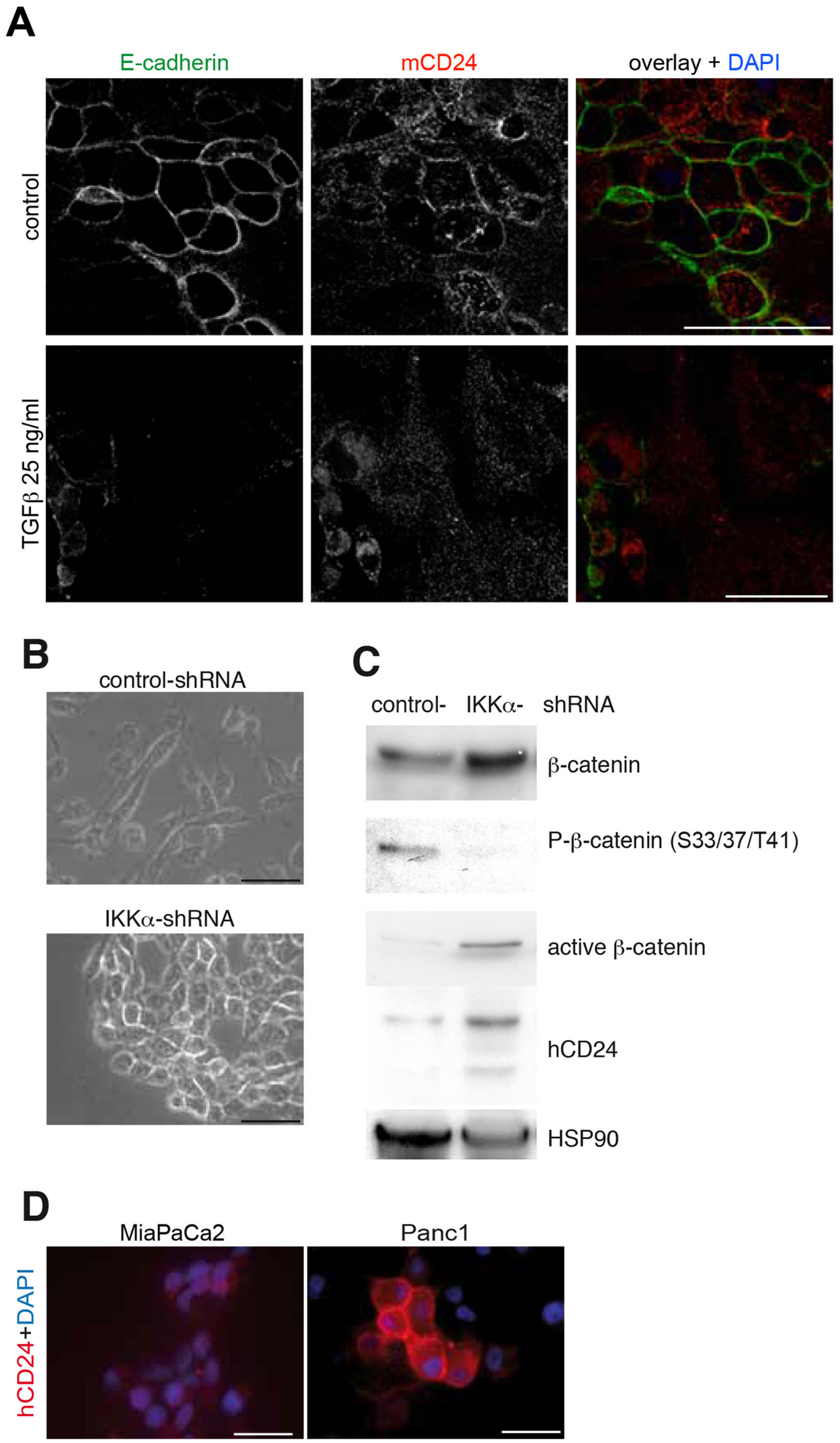

Supplementary Figure S6: CD24 expression during EMT. A. Murine pancreatic tumor cells were treated with TGFβ for 48 hours and analyzed by immunofluorescence staining. Downreguation of E-cadherin (green) expression indicates induction of EMT. **B.** The mesenchymal pancreatic cell line MiaPaCa2 acquires an epithelial phenotype upon stable transfection with IKKα siRNA. **C.** Western blot analysis shows upregulation of total β-catenin in concomitance with dephosphorylation of S33/37/T41. **D.** Immunofluorescence staining for CD24 in two human pancreatic cancer cell lines. Scale bars = 50 μM.
